# Effectiveness of Mindfulness-Based Cognitive Therapy for Improving Subjective and Eudaimonic Well-Being in Healthy Individuals: A Randomized Controlled Trial

**DOI:** 10.3389/fpsyg.2021.700916

**Published:** 2021-08-27

**Authors:** Teppei Kosugi, Akira Ninomiya, Maki Nagaoka, Zenta Hashimoto, Kyosuke Sawada, Sunre Park, Daisuke Fujisawa, Masaru Mimura, Mitsuhiro Sado

**Affiliations:** ^1^Department of Neuropsychiatry, Keio University School of Medicine, Tokyo, Japan; ^2^Department of Psychiatry, Gunma Hospital, Takasaki, Japan; ^3^Center for Stress Research, Keio University, Tokyo, Japan; ^4^Research Center for Child Mental Development, Chiba University, Chiba, Japan; ^5^Faculty of Nursing and Medicine Care, Keio University, Tokyo, Japan; ^6^Palliative Care Center, Keio University Hospital, Tokyo, Japan; ^7^Division of Patient Safety, Keio University Hospital, Tokyo, Japan

**Keywords:** subjective well-being, mindfulness, mindfulness based cognitive therapy, healthy volunteers, quality of life, resilience, happiness, eudaimonic well-being

## Abstract

**Objectives:** Better subjective and eudaimonic well-being fosters better health conditions. Several studies have confirmed that mindfulness-based interventions are effective for improving well-being; however, the samples examined in these studies have been limited to specific populations, and the studies only measured certain aspects of well-being rather than the entire construct. Additionally, few studies have examined the effect of mindfulness-based cognitive therapy on well-being. The present study examines the feasibility of mindfulness-based cognitive therapy and its effectiveness for improving subjective and eudaimonic well-being among community residents.

**Methods:** The study design featured an 8-week randomized, waiting-list controlled, parallel-group study. 8 weekly mindfulness classes, followed by 2 monthly classes, were provided for healthy individuals aged 20–65 years who had a Satisfaction with Life Scale score of ≤ 24 indicating average to low cognitive aspect of subjective well-being. This trial was registered with the University Hospital Medical Information Network Clinical Trials Registry (ID: UMIN000031885, URL: https://upload.umin.ac.jp/cgi-open-bin/ctr_e/ctr_view.cgi?recptno=R000036376).

**Results:** The results showed that cognitive aspect of subjective well-being and mindfulness skills were significantly improved at 8 weeks, and this effect was enhanced up to the end of the follow-up period. Positive affective aspect of subjective and eudaimonic well-being were significantly improved at 16 weeks.

**Conclusions:** Eight weeks of mindfulness-based cognitive therapy with a 2-month follow-up period improves cognitive and affective aspects of subjective and eudaimonic well-being in healthy individuals. The order of improvement was cognitive, positive affective, and eudaimonic well-being. To verify these findings, multi-center randomized controlled trials with active control groups and longer follow-up periods are warranted.

## Introduction

Research on well-being has been gaining increasing academic attention recently. (Ryff, 2014; Diener et al., 2018). In this field, subjective well-being (SWB) and eudaimonic well-being are the two dominant concepts (Keyes et al., 2002; Ryff, 2014; Diener et al., 2018), which differ in the way they approach well-being (Keyes et al., 2002). SWB represents a global assessment of how a person evaluates his or her own life and experiences (Diener, 1984). Two aspects of SWB have been identified, namely, life evaluation, and affect (OECD, 2013; Diener et al., 2018). Life evaluation relates to the cognitive aspect of SWB, and represents a person's satisfaction with their life (Diener et al., 1985). The affective aspect of SWB relates to the experience of both positive and negative emotions (OECD, 2013). Eudaimonic well-being is a type of well-being that has been discussed in humanistic, existential, developmental, and clinical psychology since the philosophy of Aristotle in ancient Greece, which represents a person's level of functioning and realization of their potential (Keyes et al., 2002; Huppert et al., 2009; OECD, 2013; Ryff, 2014). The cognitive and affective aspects of SWB and eudaimonic well-being are mildly correlated, but are clearly distinguishable from one another (Keyes et al., 2002; OECD, 2013; Diener et al., 2018). When measuring an individual's well-being, taking into account both SWB and eudaimonic well-being can provide a comprehensive snapshot of their well-being (OECD, 2013).

Better status in each type of well-being contributes to better health (Diener et al., 2018), including lower stress (Diener et al., 2017), higher resilience (Diener et al., 2018), and longer life expectancy (Howell et al., 2007; Diener and Chan, 2011; Steptoe et al., 2015). Therefore, from a public health perspective, developing intervention methods to improve the well-being of the general public is very important.

Some interventions, such as keeping positive events diaries (Burton and King, 2004; Lyubomirsky et al., 2011), expressing gratitude (Lyubomirsky et al., 2011), and performing acts of kindness (Buchanan and Bardi, 2010) are effective for improving well-being in both clinical and non-clinical settings (Bolier et al., 2013). In addition, mindfulness-based interventions (MBIs) have shown potential to improve well-being among the general public.

Although the original target of MBIs was clinical populations such as people with depression (Teasdale et al., 2000), anxiety disorders (Hoge et al., 2013), patients with cancer who were experiencing psychological distress (Foley et al., 2010), and people experiencing chronic pain (Khoury et al., 2013), MBIs have also been applied outside of the medical field, including to healthy populations, targeting stress management or well-being improvement. Mindfulness-to-meaning theory is one theory that explains the mechanism of the effects of mindfulness on well-being. According to this theory, the improvement of metacognitive awareness through mindfulness training promotes a positive reappraisal of experiences. As a result, the increasing positivity creates a sense of meaning in life, which enhances eudaimonic well-being (Garland et al., 2015). Several other studies have also confirmed that MBIs are effective for mitigating stress and improving well-being (Shapiro et al., 1998, 2005, 2011; Cohen-Katz et al., 2005; Jain et al., 2007; Carmody and Baer, 2008; Vieten and Astin, 2008; Klatt et al., 2009; Robins et al., 2012; de Vibe et al., 2013; Flook et al., 2013; Malarkey et al., 2013; Nyklicek et al., 2013; Huang et al., 2015; Song and Lindquist, 2015; Ivtzan et al., 2016, 2018; van Dongen et al., 2016; Bartlett et al., 2017; Chu and Mak, 2020).

However, there are some limitations to the above findings. First, they targeted specific populations such as students (Shapiro et al., 1998, 2011; Jain et al., 2007; de Vibe et al., 2013; Song and Lindquist, 2015), school teachers (Klatt et al., 2009; Flook et al., 2013), health-care workers (Cohen-Katz et al., 2005; Shapiro et al., 2005), and employees in their workplaces (Malarkey et al., 2013; Huang et al., 2015; van Dongen et al., 2016; Bartlett et al., 2017). It is therefore difficult to generalize these results to the community. Second, neither of the two studies (Robins et al., 2012; Nyklicek et al., 2013) that targeted healthy people in the community assessed both SWB and eudaimonic well-being simultaneously. Consequently, it is unclear how MBIs affect each aspect of these well-beings. Finally, despite the confirmed effects of mindfulness-based stress reduction (MBSR) on well-being (Khoury et al., 2015), the evidence of mindfulness-based cognitive therapy (MBCT), another major MBI (Segal et al., 2002), on well-being is sparse. Considering these limitations, our study aims to perform a randomized controlled trial (RCT) to assess the feasibility and effectiveness of MBCT in improving the cognitive and affective aspects of subjective and eudaimonic well-being among healthy people enrolled from a community sample. The economic evaluation will be reported separately.

## Materials and Methods

This study was performed at Keio University Hospital, Tokyo, Japan. The study protocol is published (Sado et al., 2020) and was registered in the University Hospital Medical Information Network Clinical Trials Registry (UMIN000031885; https://upload.umin.ac.jp/cgi-open-bin/ctr_e/ctr_view.cgi?recptno=R000036376) and was performed and reported according to CONSORT guidelines.

### Participants

Participants were recruited through the Center for Stress Research at Keio University (Keio CSR). Eligibility criteria were healthy individuals who (1) were aged 20–65 years, (2) had no history of psychiatric disorder in the past 2 years, (3) scored ≤ 24 on the Satisfaction With Life Scale (SWLS) (Diener et al., 1985) indicating average to low cognitive aspect of subjective well-being (Oishi, 2009), and (4) were able to complete a consent form.

The exclusion criteria were (1) being likely to be difficult to follow up, (2) previous engagement in MBIs similar to that administered in this study, and (3) having a severe physical illness.

### Enrollment

Participants applied through a form presented on Keio CSR's website and answered online screening questionnaires (first screening). Each participant who passed the first screening was then interviewed by a psychiatrist or psychologist from the study team (second screening) to confirm whether they satisfied the eligibility criteria. For diagnostic assessment, participants were screened using the Japanese version of the Structured Clinical Interview for DSM-IV Axis I Disorders (American Psychiatric Association, 2000). Participants were enrolled according to the results of the second screening. Eligible participants were given detailed explanations of the study procedures, after which they provided written informed consent.

### Baseline Assessment

A battery of questionnaires was used to collect demographic and psychosocial data from the eligible participants. Details of the psychological measures are provided below.

### Randomization and Masking

All enrolled participants were randomly assigned 1:1 to the MBCT group or the waiting list control group (control group) with masking for researchers and participants. Assignment was performed using a computer-generated random number system that was administered independently of the present authors by the Keio Center of Clinical Research Project Management Office. Randomization was stratified using the participants' baseline scores for the SWLS (≥20, ≤19). The nature of the psychological intervention in question meant that the allocation group of the participants and intervention instructors could not be blinded. As all outcome instruments utilized were self-reported, participants were not assessed by anyone.

### Interventions

#### MBCT Group

The intervention program comprised a modified version of MBCT, and was based on the guide developed by Williams and Penman (2011). MBI programs need to be based on MBCT and MBSR, and adjusted appropriately for the target of intervention (Crane et al., 2017). As the MBCT was originally developed for patients with depression (Teasdale et al., 2000), for the present study, we modified the MBCT program to focus on improving well-being of a non-clinical population. The contents of the program are shown in Table 1. The main differences between our program and the original MBCT were as follows: (1) a psychoeducational lecture concerning depression was omitted from our program, and (2) compassion meditation and activity records (pleasant, unpleasant, appreciation events, and nourishing and depriving activities) were added to the program. Through the program, participants experienced mindfulness practices (e.g., the raisin exercise, body scan, sitting meditation, mindful walking, and three-step breathing space) and learned cognitive approaches.

**Table 1:** Contents of the program.

**Session**	**Theme**	**Contents**
1	Waking up to the automatic pilot	Psychoeducation: What is mindfulness?
		Exercise: Mindfulness eating (‘raisin exercise’)/asking yourself why you are here now/mindfulness of body and breath
		Homework: Mindfulness of body and breath/mindfulness of a routine activity/Let go of habits
2	Keeping the body in mind	Psychoeducation: Association of mood and thoughts
		Exercise: Mindfulness of body and breath/ Thoughts and feelings exercise/Body scan
		Homework: Body scan/pleasant event calendar/mindfulness in everyday life/Let go of habits
3	The mouse in the maze	Psychoeducation: Awareness of mind wandering and focusing on the breath
		Exercise: Breathing meditation/meditation of sounds/gentle yoga/mindful walking
		Homework: Three-minute breathing space/gentle yoga/mindful walking/Diary of appreciation and gratitude events/let go of habits
4	Moving beyond the rumor mill	Psychoeducation: Staying present
		Exercise: Mindfulness meditations (breathing/sounds and thoughts)
		Homework: Mindfulness meditations (breathing/sounds and thoughts/three-minute breathing space)/Unpleasant events calendar/Let go of habits
5	Turning toward difficulties	Psychoeducation: Exploring difficulty
		Exercise: Mindfulness meditations (breathing/sounds and thoughts/exploring difficulty)
		Homework: Mindfulness meditations (breathing/sounds and thoughts/exploring difficulty/three-minute breathing space)/Let go of habits
6	Trapped in the past or living in the present	Psychoeducation: Cognitive biases/Compassion for myself
		Exercise: Mindfulness meditations/compassion meditation/watching the movie “Happy” about well-being.
		Homework: Mindfulness meditations (sounds and thoughts/exploring difficulty/compassion/three-minute breathing space)/diary of your kind behaviour
7	When did you stop dancing	Psychoeducation: Choosing functional behaviours/behavioural activation/identifying triggers
		Exercise: Mindfulness meditations (breathing/sounds and thoughts)
		Homework: Mindfulness meditations (choose what you like/three-minute breathing space)/diary of activity that nourishes
8	Your wild and precious life	Psychoeducation: Personal reflections of course/plans for future practice and strategies for maintaining momentum/farewell
		Exercise: Body scan/asking yourself why you are here now and what you realised through the program

Eight 2-h sessions, administered once a week, were implemented in the program. Each session was conducted in groups (of up to 15 participants). Once these eight sessions were completed, two booster sessions, once a month, were provided during a 2-month follow-up period. During the initial 8-week period, participants were given daily homework in which they performed mindfulness meditation for 30–60 min. No homework was assigned during the follow-up period. Instead, participants were asked to submit a short essay each month regarding their daily practice to share their experiences with the other group members.

#### Control Group

During the intervention period, no interventions were provided for the participants on the waiting list. These participants were instructed not to participate in other mindfulness or meditation activities during this period. Once the intervention period had ended, the control group was given opportunities to participate in the MBCT program.

### Outcomes

#### Primary Outcome

The primary outcome was the difference between the MBCT group and control group regarding their respective mean change scores for SWLS from the baseline evaluation to the post-intervention evaluation.

#### Secondary Outcomes

The secondary outcomes were the differences between the MBCT group and control group regarding their respective mean change scores (again from the baseline to the post-intervention assessment) for the variables measured using the instruments listed in the following section.

### Instruments

#### Satisfaction With Life Scale

The SWLS is a self-reported questionnaire that assesses life satisfaction through five questions. Total score is in the range of 5 to 35, and higher scores represent increased cognitive aspect of subjective well-being (Diener et al., 1985).

#### Flourishing Scale

The Flourishing Scale (FS) comprises eight items measuring factors that represent the social-psychological well-being linked to eudaimonic well-being to complement existing measures of SWB. It can measure well-being in eight dimensions that are based on existing theories plus recent theories related to psychological and social well-being. Total score is in the range of 8 to 56. Higher scores indicate higher eudaimonic well-being (Diener et al., 2010).

#### Scale of Positive and Negative Experience

The Scale of Positive and Negative Experience (SPANE) is a 12-item scale (six items assess positive experiences and six assess negative experiences) that measures the affective aspect of SWB. The scale evaluates the whole range of positive and negative experiences with specific feelings. The positive and negative scales are scored separately because the two types of emotions are partially independent or separable. The positive score (SPANE-P) and the negative scale (SPANE-N) are in the range of 6 to 30. Higher scores represent higher positive or negative affective aspects of SWB. Subtracting the negative score from the positive score represents the SPANE-B score that ranges from −24 to 24 (Diener et al., 2010).

#### Rosenberg Self-Esteem Scale

The Rosenberg Self-Esteem Scale (RSES) is a self-administered rating scale that assesses self-esteem. Total score is in the range of 10 to 40. Higher scores indicate higher self-esteem (Rosenberg, 1965).

#### Five Facet Mindfulness Questionnaire

The Five Facet Mindfulness Questionnaire (FFMQ) is a self-reported questionnaire that assesses mindfulness abilities. The five facets are observing, describing, acting with awareness, non-judging, and non-reacting. Higher scores indicate higher mindfulness abilities (Baer et al., 2006).

#### Connor Davidson Resilience Scale

The Connor Davidson Resilience Scale (CDRISC) is a self-reported scale measuring resilience over the past month. Total score is in the range of 0 to 100, with higher scores indicating greater resilience (Connor and Davidson, 2003).

#### Self-Compassion Scale

The Self-Compassion Scale (SCS) measures a person's ability to understand themself with kindness, rather than being harsh or self-critical of their pain and failure. It comprises 29 items and six subscales (self-kindness, self-judgment, common humanity, isolation, mindfulness, and over-identification); scores for each subscale range from 1 to 5 and are calculated from the average score of each component. The sum of the subscales is the total SCS scale score. Its range is from 5 to 30, with higher scores indicating more self-compassion (Neff, 2003).

#### 16-Item Quick Inventory of Depressive Symptomatology

The 16-item Quick Inventory of Depressive Symptomatology (QIDS) is a self-reported questionnaire that assesses depressive symptoms. The total score is in the range of 0 to 27. Higher scores represent increased depression (Rush et al., 2003).

#### General Anxiety Disorder-7

The General Anxiety Disorder-7 (GAD-7) examines anxiety symptoms experienced during the previous 2 weeks. Total score is in the range of 0 to 21. Higher scores represent higher levels of anxiety symptoms (Spitzer et al., 2006).

#### Perceived Stress Scale

The Perceived Stress Scale (PSS) evaluates stress regarding one's life conditions over the previous month. Total score is in the range of 0 to 40. Higher scores indicate higher stress levels (Cohen et al., 1983).

#### World Health Organization Health and Work Performance Questionnaire

The World Health Organization Health and Work Performance Questionnaire (WHO-HPQ) is a self-reported scale that evaluates the workplace costs of health issues relating to loss of job performance (presenteeism). The scale has two items, one to assess one's own work performance and the other to assess how well one's colleagues are performing at work. Both items have a range of 0 to 10. Absolute presenteeism is a ten-fold increase in the former item's score (range from 0 to 100). Relative presenteeism is the score of the former divided by the score of the latter (range from 0.25 to 2.0). Higher scores indicate a higher rating for work performance (Kessler et al., 2003).

#### Multidimensional Assessment of Interceptive Awareness

Interceptive awareness is known to be a vital element in meditation and stress reduction (Bornemann et al., 2015) The Multidimensional Assessment of Interceptive Awareness (MAIA) is a 32-item instrument that assesses interceptive awareness using the following eight dimensions: noticing, not-distracting, not-worrying, attention regulation, emotional awareness, self- regulation, body listening, and trusting. Total scores for each dimension are in the range of 0 to 5 (Mehling et al., 2012).

#### EuroQoL-5 Dimensions 5-Level

The EuroQoL-5 Dimensions 5-level (EQ-5D-5L) measures health outcomes and quality of life. It is a five-item questionnaire that focuses on health-related quality of life. Total score is in the range of 0 to 1 (Herdman et al., 2011).

### Instrument Reliability and Validity

The reliability and validity of all of the above instruments have been proven for Japanese populations (Kadono, 1994; Hamashima and Yoshida, 2001; Tsuchiya et al., 2002; Mimura and Griffiths, 2007, 2008; Ito et al., 2009; Muramatsu et al., 2009, 2010; Fujisawa et al., 2010; Sugiura et al., 2011; Arimitsu, 2014; Sumi, 2014; Doi et al., 2018; Shoji et al., 2018; Kawakami et al., 2020).

### Schedule of Visits and Assessments

All participants completed these instruments at baseline, 4, 8 weeks, and at the end of the follow-up. The schedule of assessment is shown in Table 2.

**Table 2:** Schedule of assessments.

	**Screening**		**Week**										
	**s-1**	**s-2**	**0**	**1**	**2**	**3**	**4**	**5**	**6**	**7**	**8**	**12**	**16**
Screening (web)	◯												
Screening (face to face interview)		◯											
Informed consent			◯										
Randomization			◯										
MBCT Class				◯	◯	◯	◯	◯	◯	◯	◯	◯	◯
SCID		◯											
Demographics	◯		◯										
SWLS			◯				◯				◯		◯
FS			◯				◯				◯		◯
SPANE			◯				◯				◯		◯
RSES			◯				◯				◯		◯
FFMQ			◯				◯				◯		◯
CDRISC			◯				◯				◯		◯
SCS			◯				◯				◯		◯
QIDS			◯				◯				◯		◯
GAD7			◯				◯				◯		◯
PSS			◯				◯				◯		◯
WHO-HPQ			◯				◯				◯		◯
MAIA			◯				◯				◯		◯
EQ-5D-5L			◯				◯				◯		◯

*SCID, Structured Clinical Interview for DSM-IV; SWLS, Satisfaction With Life Scale; FS, Flourishing Scale; SPANE, Scale of Positive and Negative Experience; RSES, Rosenberg Self-Esteem Scale; FFMQ, Five Facet Mindfulness Questionnaire; CDRISC, The Connor Davidson; RS, Resilience Scale; SCS, Self-Compassion Scale; QIDS, The 16-item Quick Inventory of Depressive Symptomatology; GAD7, 7-item anxiety scale; PSS, Perceived Stress Scale; WHO-HPQ, The World Health Organization Heath and Work Performance Questionnaire; MAIA, The Multidimensional Assessment of Interceptive Awareness; EQ-5D-5L, Five level EuroQoL-5 Dimensions. ◯ symbol means that an assessment, intervention, randomization, informed consent, or interview was conducted at that time*.

### Sample Size

We conducted a pilot study (unpublished) to explore the feasibility, safety, and effectiveness of MBCT for improving well-being before this study. The pilot study showed that the mean SWLS score had a pre/post difference of 3.1 (SD = 3.4). Based on the results, a sample size calculation was performed to obtain a statistical power of at least 80% and a significance level of 5% (two-sided), and it was determined that 20 participants were required in each group. Estimating a dropout rate of 20%, 25 participants in each arm were needed (50 participants in total).

### Statistical Analysis

To compare the two groups' baseline demographic and clinical characteristics, unpaired *t*-tests were adopted for the continuous variables and chi-square tests for the categorical variables. The primary and secondary outcomes were analyzed using an intention-to-treat approach, and a mixed-effects model repeat measurement was used to control for dropouts. A fixed-effects model was used for the intervention group, week, and group-by-week interaction, age, and sex. A 5% significance level was used for all statistical analyses. Stata Version 14 software (StataCorp LLC, College Station, TX, USA) was adopted to perform the statistical analyses. For well-being outcomes (i.e., SWLS, FS, and SPANE), the effect size was calculated dividing the difference between groups by its standard deviation.

## Results

**Figure 1:**
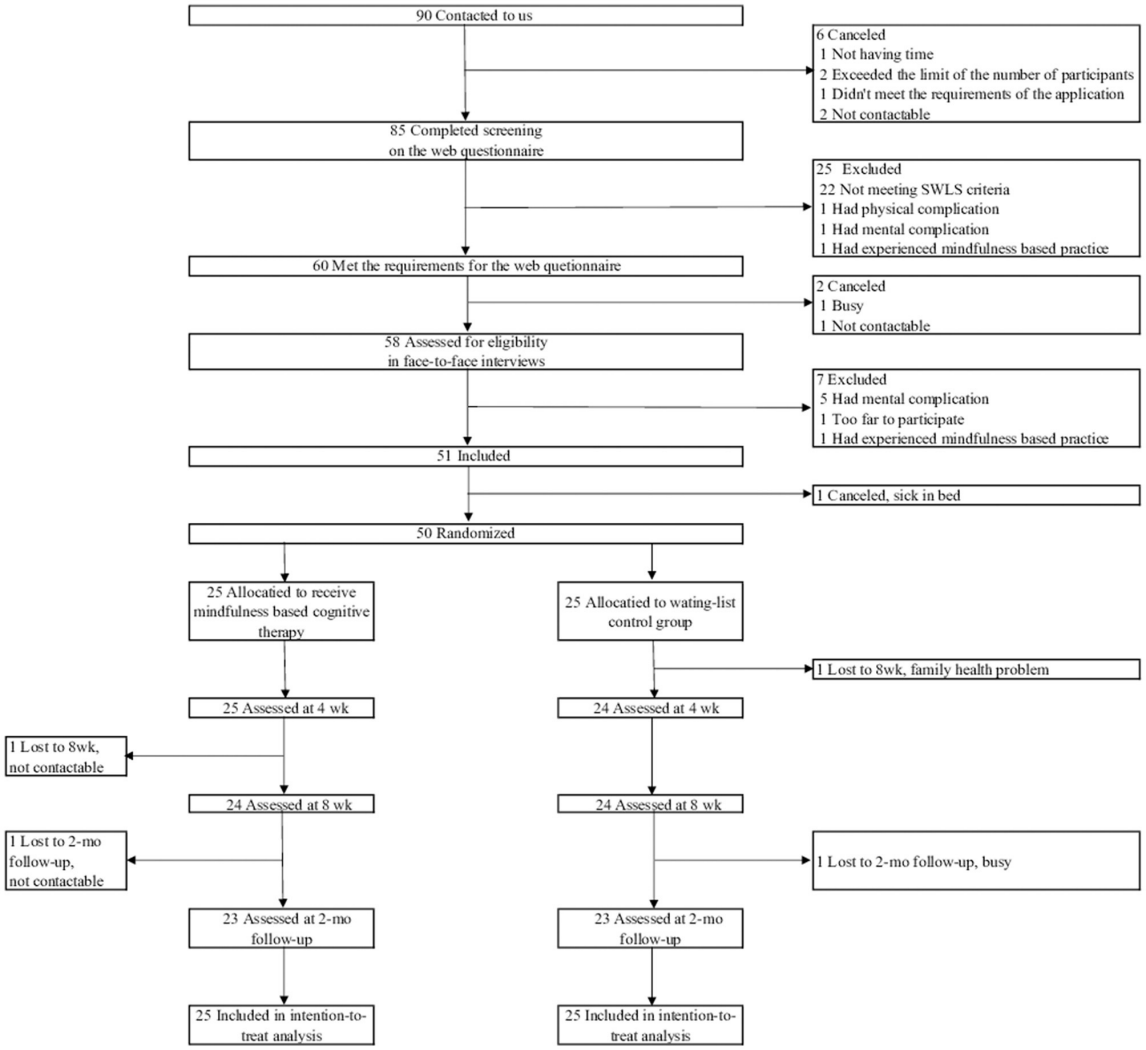
Flow diagram of the study.

The flow of the study from screening to post intervention is shown in Figure 1. This study began recruiting participants in July 2018 and final data collection was completed in December 2019. Dataset construction was completed at the end of March 2020. Beginning in July 2018, 90 participants were screened. Fifty participants (55.6%) met the inclusion criteria and were randomly allocated to the MBCT group (*n* = 25) or the waiting-list control group (*n* = 25). Two participants in each group dropped out. The participants' baseline demographic and clinical characteristics are shown in Tables 3, 4. The average age was 46.8 ± 8.7 years, and 78.0% were female. All participants were employed or were students or homemakers, and the average household income was relatively high. No significant differences between the groups were observed with respect to sociodemographic status. In terms of the clinical measures, there were no significant differences, except for SPANE-P, SPANE-B and MAIA Not-Worrying.

**Table 3:** Baseline sociodemographic characteristics.

**Sociodemographic characteristics**	**Total** **(** ***n*** **= 50)**		**MBCT** **(** ***n*** **= 25)**		**Control** **(** ***n*** **= 25)**		**Statistics**
Age, mean (SD), y	46.8	(8.7)	47.8	(9.6)	45.7	(7.8)	0.39^a^
Sex (female), *n* (%)	39	(78.0)	20	(80.0)	19.0	(76.0)	0.73^a^
Education, *n* (%),							0.44^b^
High school	3	(6.0)	2	(8.0)	1	(4.0)	
Junior college/vocational school	12	(24.0)	4	(16.0)	8	(32.0)	
University	22	(44.0)	13	(52.0)	9	(36.0)	
Graduate school	12	(24.0)	5	(20.0)	7	(28.0)	
Unclear	1	(2.0)	1	(4.0)	0	(0.0)	
Job, *n* (%)							0.51^b^
President/exective	6	(12.0)	1	(4.0)	5	(20.0)	
Office worker	16	(32.0)	6	(24.0)	10	(40.0)	
Civil servant	3	(6.0)	2	(8.0)	1	(4.0)	
Self-employed	7	(14.0)	5	(20.0)	2	(8.0)	
Freelancer	1	(2.0)	1	(4.0)	0	(0.0)	
Profession	6	(12.0)	3	(12.0)	3	(12.0)	
Part-time worker	4	(8.0)	2	(8.0)	2	(8.0)	
Student	2	(4.0)	1	(4.0)	1	(4.0)	
Homemaker	4	(8.0)	3	(12.0)	1	(4.0)	
Others	1	(2.0)	1	(4.0)	0	(0.0)	
Marital status, *n* (%)							0.79^b^
Married	30	(60.0)	15	(60.0)	15	(60.0)	
Single	15	(30.0)	7	(28.0)	8	(32.0)	
Separated, divorced,	4	(8.0)	2	(8.0)	2	(8.0)	
Widowed	1	(2.0)	1	(4.0)	0	(0.0)	
Cohabiting, *n* (%)	35	(70.0)	17	(68.0)	18	(72.0)	0.76^a^
Physical complications, *n* (%)	8	(16.0)	3	(12.0)	5	(20.0)	0.44^a^
Household income (yen), *n* (%)							0.97^b^
<2,000,000	1	(2.0)	1	(4.0)	0	(0.0)	
2,000,000 ≤, 4,000,000 <	2	(4.0)	1	(4.0)	1	(4.0)	
4,000,000 ≤, 6,000,000 <	9	(18.0)	5	(20.0)	4	(16.0)	
6,000,000 ≤, 8,000,000 <	6	(12.0)	3	(12.0)	3	(12.0)	
8,000,000 ≤, 10,000,000 <	7	(14.0)	3	(12.0)	4	(16.0)	
10,000,000 ≤, 12,000,000 <	7	(14.0)	3	(12.0)	4	(16.0)	
12,000,000 ≤	18	(36.0)	9	(36.0)	9	(36.0)	
Income (yen), *n* (%)							0.67^b^
<2,000,000	11	(22.0)	8	(32.0)	3	(12.0)	
2,000,000 ≤, 4,000,000 <	8	(16.0)	4	(16.0)	4	(16.0)	
4,000,000 ≤, 6,000,000 <	11	(22.0)	4	(16.0)	7	(28.0)	
6,000,000 ≤, 8,000,000 <	9	(18.0)	5	(20.0)	4	(16.0)	
8,000,000 ≤, 10,000,000 <	3	(6.0)	1	(4.0)	2	(8.0)	
10,000,000 ≤, 12,000,000 <	3	(6.0)	1	(4.0)	2	(8.0)	
12,000,000 ≤	5	(10.0)	2	(8.0)	3	(12.0)	

a *Student's t-test*.

b *Chi-square test*.

**Table 4:** Baseline clinical outcomes.

**Measures**	**Total** **(*n* = 50)**		**MBCT** **(** ***n*** **= 25)**		**Control** **(** ***n*** **= 25)**		**Statistics^a^**
	**Mean**	**SD**	**Mean**	**SD**	**Mean**	**SD**	
SWLS	18.5	4.3	18.7	4.2	18.3	4.5	0.75
FS	39.0	6.0	40.6	5.1	37.5	6.5	0.06
SPANE-P	22.9	3.1	21.3	2.8	18.8	2.8	0.003
SPANE-N	15.8	4.0	17.0	3.6	18.6	2.9	0.09
SPANE-B	2.3	5.0	4.3	5.4	0.20	3.6	0.00
FFMQ total^b^	118.3	14.3	119.8	13.1	116.7	15.5	0.45
MAIA							
Noticing	2.7	0.90	2.8	0.98	2.7	0.84	0.59
Not-distracting	2.6	1.0	2.4	0.91	2.8	1.1	0.16
Not-worrying	2.4	0.85	2.7	0.63	2.2	0.96	0.03
Attention regulation	2.7	0.59	2.8	0.55	2.7	0.62	0.47
Emotional awareness	2.8	0.87	2.9	0.86	2.7	0.88	0.52
Self-Regulation	2.8	0.81	2.9	0.76	2.7	0.86	0.39
Body listening	2.0	1.2	2.2	0.99	1.8	1.3	0.19
Trusting	2.4	0.94	2.6	0.90	2.2	0.93	0.09
RSES	23.8	3.6	23.2	3.6	24.3	3.6	0.68
CDRISC	59.0	14.1	62.6	13.1	55.4	14.4	0.07
SCS total	17.6	3.3	18.2	2.9	17.0	3.6	0.21
PSS^*b*^	17.8	4.1	16.8	3.9	18.8	4.1	0.08
GAD7	4.3	3.1	4.2	3.4	4.3	2.9	0.93
QIDS	3.8	3.0	3.6	2.5	4.0	3.5	0.68
EQ-5D-5L utility	0.90	0.11	0.92	0.11	0.88	0.11	0.81
WHO-HPQ (absolute presenteeism)^c^	55.8	21.3	55.2	25.4	56.5	16.4	0.83
WHO-HPQ (relative presenteeism)^c^	0.99	0.30	0.92	0.31	1.1	0.27	0.10

a *Student's t-test*.

b *Total number decreased in the Control group (n=25 → 24, FFMQ, PSS) because of missing value*.

c *Exclude those not working; results for 48 in total, 25 in the MBCT group and 23 in the Control group*.

*CD-RISC, the Connor-Davidson Resilience Scale; EQ-5D-5L, Five level EuroQoL-5 Dimensions; FFMQ, Five Facet Mindfulness Scale; FS, Flourishing Scale; GAD7, Generalized Anxiety Disorder Questionnaire; MAIA, The Multidimensional Assessment of Interoceptive Awareness; PSS, Perceived Stress Scale; QIDS, Quick Inventory of Depressive Symptomatology; RSES, Rosenberg Self Esteem Scale; SCS, Self-Compassion Scale; SPANE, Scale of Positive and Negative Experience; SWLS, Satisfaction With Life Scale; WHO-HPQ, The World Health Organization Heath and Work Performance Questionnaire*.

Treatment engagement is shown in Table 5. Average attendance of the 8-week session was 7.08 ± 1.58 classes. Fourteen (56.0%) participants completed the 8-week session. The average attendance of the 8 weekly sessions plus the two follow-up classes was 8.76 ± 2.05 classes. No serious adverse events were observed over the study period. Total mean homework time during the 8-week session was 1202.2 ± 455.5 min.

**Table 5:** Treatment engagement.

**Variable**	**MBCT (** ***n*** **= 25)**	
8 week session, *n* (SD) (Weekly, range: 0–8)	7.08 (1.58)	
Completion rate of the full course of MBCT sessions (8 week), *n* (%)	14.0 (56.0)	
Follow up session, *n* (SD) (Monthly, range: 0–2 sessions).	1.68 (0.63)	
Total, *n* (SD) (Range: 0–10 sessions)	8.76 (2.05)	
Home work time (min)	1202.2 (455.5)	

### Primary Outcome

Differences between the MBCT group and the control group regarding mean change scores for SWLS scores (effect size: 0.47; difference: 2.79; 95% confidence interval [CI]: 0.39–5.20; *P* = 0.023) were significant at 8 weeks (Table 6). The MBCT's effect was even stronger at the 2-month follow-up (effect size: 0.55; difference: 3.38; 95% CI: 0.94–5.81; *P* = 0.007).

**Table 6:** Effects of repeated-measure analyses of well-being outcomes (intention-to-treat population).

**Measures**	**Time points**	**MBCT**		**Control**		**Difference in mean change scores (95% Cl)**		***P* Value**	**Effect size**
		**(** ***n*** **= 25)**		**(** ***n*** **= 25)**					
		**Mean**	**SD**	**Mean**	**SD**				
SWLS	Baseline	18.7	4.2	18.3	4.5				
	4-wk	21.0	4.4	19.5	5.4	0.90	(−1.49 to 3.28)	0.46	0.21
	8-wk	22.8	5.3	19.6	5.9	2.79	(0.39 to 5.20)	0.02	0.47
	2-mo follow-up	24.3	4.9	20.5	5.4	3.38	(0.94 to 5.81)	0.007	0.55
FS	Baseline	40.6	5.1	37.5	6.5				
	4-wk	42.4	5.1	37.1	7.4	2.07	(−0.45 to 4.60)	0.11	0.63
	8-wk	43.2	5.3	38.7	5.3	1.39	(−1.15 to 3.93)	0.28	0.57
	2-mo follow-up	45.0	7.2	38.4	5.6	3.56	(0.98 to 6.14)	0.007	0.81
SPANE-P	Baseline	21.3	2.8	18.8	2.8				
	4-wk	22.8	2.7	20.0	3.2	0.08	(−1.57 to 1.73)	0.92	0.78
	8-wk	23.1	3.0	19.3	3.1	1.24	(−0.41 to 2.90)	0.14	0.96
	2-mo follow-up	24.4	3.3	19.1	3.4	2.89	(1.21 to 4.57)	0.001	1.33
SPANE-N	Baseline	17.0	3.6	18.6	2.9				
	4-wk	15.2	3.6	17.0	4.2	−0.40	(−2.41 to 1.60)	0.70	0.39
	8-wk	15.4	4.3	17.8	3.5	−1.04	(−3.06 to 0.97)	0.31	0.50
	2-mo follow-up	15.7	4.5	17.1	3.6	−0.08	(−2.13 to 1.96)	0.94	0.31
SPANE-B	Baseline	4.3	5.4	0.2	3.6				
	4-wk	7.6	5.1	3.0	6.1	0.47	(−2.59 to 3.52)	0.76	0.65
	8-wk	7.7	6.3	1.5	5.6	2.27	(−0.81 to 5.34)	0.15	0.86
	2-mo follow-up	8.7	7.0	2.0	5.9	2.95	(−0.16 to 6.07)	0.06	0.91

*FS, Flourishing Scale; SPANE, Scale of Positive and Negative Experience; SWLS, Satisfaction With Life Scale*.

### Secondary Outcomes

Scores for the FS and SPANE-P (which represented positive affect) showed significant improvement at the 2-month follow-up (Table 6). There was a significant improvement in total FFMQ, SCS and PSS score at 8 weeks, and in CDRISC scores and in WHO-HPQ absolute presentism at 2 months (Table 7). For the MAIA subscales, there were significant improvements in Noticing, Not-worrying, Attention regulation, Emotional awareness, Self-regulation, Body listening, and Trusting at 4 weeks, and in Not-distracting at 8 weeks (Table 7). No significant differences were found for SPANE-N, SPANE-B, GAD, QIDS, EQ-5D-5L utility, or WHO-HPQ relative presentism (Tables 6, 7).

**Table 7:** Summary of repeated-measure analyses of other outcomes (intention-to-treat population).

**Clinical measures**	**Time points**	**MBCT**		**Control**		**Difference in mean change scores (95% Cl)**	***P* Value**
		**(** ***n*** **= 25)**		**(** ***n*** **= 25)**			
		**Mean**	**SD**	**Mean**	**SD**		
FFMQ (total)	Baseline	119.8	13.1	116.7	15.5		
	4-wk	127.5	14.5	120.4	15.0	3.46 (−2.57 to 9.49)	0.26
	8-wk	131.6	14.4	118.6	16.4	9.86 (3.70 to 16.03)	0.002
	2-mo follow-up	138.6	13.5	120.3	15.8	14.82 (8.56 to 21.08)	<0.001
MAIA
Noticing	Baseline	2.79	0.98	2.65	0.84		
	4-wk	3.14	0.82	2.16	0.92	0.87 (0.39 to 1.35)	<0.001
	8-wk	3.38	1.11	2.26	0.91	1.01 (0.53 to 1.50)	<0.001
	2-mo follow-up	3.52	0.92	2.17	0.87	1.26 (0.77 to 1.75)	<0.001
Not-Distracting	Baseline	2.43	0.91	2.84	1.14		
	4-wk	2.67	1.02	2.57	1.14	0.50 (−0.07 to 1.08)	0.09
	8-wk	2.93	0.82	2.67	0.76	0.66 (0.09 to 1.24)	0.02
	2-mo follow-up	2.87	0.85	2.80	1.10	0.43 (−0.15 to 1.02)	0.15
Not-Worrying	Baseline	2.68	0.63	2.17	0.96		
	4-wk	2.69	0.77	2.25	0.77	0.63 (0.20 to 1.06)	0.004
	8-wk	2.69	0.69	2.36	0.65	0.10 (−0.33 to 0.53)	0.65
	2-mo follow-up	2.88	0.85	2.35	0.62	0.08 (−0.36 to 0.52)	0.72
Attention Regulation	Baseline	2.78	0.55	2.66	0.62		
	4-wk	3.18	0.61	2.46	0.77	0.62 (0.26 to 0.97)	0.001
	8-wk	3.35	0.66	2.48	0.90	0.79 (0.43 to 1.15)	<0.001
	2-mo follow-up	3.55	0.81	2.30	0.81	1.17 (0.80 to 1.53)	<0.001
Emotional Awareness	Baseline	2.90	0.86	2.74	0.88		
	4-wk	3.33	0.76	2.69	0.92	0.47 (0.07 to 0.86)	0.02
	8-wk	3.59	0.83	2.60	1.00	0.85 (0.45 to 1.24)	<0.001
	2-mo follow-up	3.75	0.77	2.43	0.92	1.17 (0.77 to 1.57)	<0.001
Self-Regulation	Baseline	2.86	0.76	2.66	0.86		
	4-wk	3.54	0.68	2.71	0.73	0.62 (0.26 to 0.98)	0.001
	8-wk	3.64	0.82	2.64	0.82	0.81 (0.45 to 1.18)	<0.001
	2-mo follow-up	3.80	0.83	2.47	0.84	1.15 (0.78 to 1.52)	<0.001
Body Listening	Baseline	2.21	0.99	1.77	1.31		
	4-wk	3.03	0.82	1.96	0.90	0.68 (0.21 to 1.15)	0.005
	8-wk	3.19	0.97	1.82	0.91	0.96 (0.48 to 1.44)	<0.001
	2-mo follow-up	3.36	1.15	2.12	1.03	0.87 (0.39 to 1.36)	<0.001
Trusting	Baseline	2.64	0.90	2.19	0.93		
	4-wk	3.39	0.71	2.07	1.01	0.89 (0.40 to 1.38)	<0.001
	8-wk	3.58	0.93	2.14	0.87	1.05 (0.55 to 1.54)	<0.001
	2-mo follow-up	3.94	0.95	2.19	1.02	1.34 (0.84 to 1.84)	<0.001
RSES	Baseline	23.2	3.6	24.3	3.6		
	4-wk	22.1	3.8	23.5	3.6	−0.23 (−1.80 to 1.34)	0.78
	8-wk	21.6	3.8	23.4	3.6	−0.79 (−2.37 to 0.79)	0.33
	2-mo follow-up	20.3	5.0	23.3	3.3	−1.97 (−3.57–0.36)	0.02
CDRISC	Baseline	62.6	13.1	55.4	14.4		
	4-wk	66.2	10.3	55.5	13.4	3.06 (−2.13 to 8.24)	0.25
	8-wk	65.8	12.7	53.9	14.1	4.88 (−0.34 to 10.10)	0.07
	2-mo follow-up	72.2	14.3	53.5	14.2	11.10 (5.80 to 16.39)	<0.001
SCS total	Baseline	18.2	2.9	17.0	3.6		
	4-wk	19.4	4.0	17.3	3.4	0.85 (−0.47 to 2.16)	0.21
	8-wk	20.3	3.8	17.5	3.4	1.58 (0.26 to 2.89)	0.02
	2-mo follow-up	21.4	4.2	17.4	3.5	2.82 (1.49 to 4.15)	<0.001
PSS	Baseline	16.8	3.9	18.8	4.1		
	4-wk	15.4	3.1	17.9	4.0	−0.09 (−2.47 to 2.30)	0.94
	8-wk	13.9	4.7	19.7	5.3	−3.46 (−5.86–1.06)	0.005
	2-mo follow-up	13.7	4.8	18.4	4.7	−2.33 (−4.76 to 0.11)	0.06
GAD7	Baseline	4.2	3.4	4.3	2.9		
	4-wk	2.6	2.1	3.5	3.1	−0.77 (−2.31 to 0.77)	0.33
	8-wk	2.5	2.6	4.0	3.6	−1.35 (−2.90 to 0.19)	0.09
	2-mo follow-up	2.3	3.0	3.2	2.0	−0.67 (−2.24 to 0.89)	0.40
QIDS	Baseline	3.6	2.5	4.0	3.5		
	4-wk	2.5	2.6	4.2	2.1	−1.27 (−2.70 to 0.16)	0.08
	8-wk	2.1	2.4	3.4	3.0	−0.90 (−2.33 to 0.53)	0.22
	2-mo follow-up	1.7	2.7	3.8	2.9	−1.44 (−2.89 to 0.00)	0.05
EQ-5D-5L utility	Baseline	0.92	0.11	0.88	0.11		
	4-wk	0.92	0.11	0.86	0.12	0.02 (−0.05 to 0.08)	0.63
	8-wk	0.94	0.10	0.87	0.11	0.03 (−0.03 to 0.10)	0.32
	2-mo follow-up	0.95	0.09	0.91	0.12	0.01 (−0.06 to 0.07)	0.85
WHO-HPQ	Baseline	60.0	20.0	56.5	16.4		
(absolute presenteeism)	4-wk	71.7	18.5	60.4	15.8	7.01 (−3.16 to 17.19)	0.18
	8-wk	71.4	18.3	58.8	16.2	9.26 (−0.91 to 19.44)	0.07
	2-mo follow-up	74.5	20.2	56.5	13.0	12.93 (2.65 to 23.21)	0.01
WHO-HPQ	Baseline	0.98	0.25	1.06	0.27		
(relative presenteeism)	4-wk	1.07	0.35	1.12	0.33	0.04 (−0.15 to 0.24)	0.66
	8-wk	1.05	0.20	1.05	0.29	0.06 (−0.14 to 0.26)	0.53
	2-mo follow-up	1.06	0.30	1.00	0.29	0.11 (−0.09 to 0.31)	0.27

*CD-RISC, the Connor-Davidson Resilience Scale; EQ-5D-5L, Five level EuroQoL-5 Dimensions; FFMQ, Five Facet Mindfulness Scale; GAD7, Generalized Anxiety Disorder Questionnaire; MAIA, The Multidimensional Assessment of Interoceptive Awareness; PSS, Perceived Stress Scale; QIDS, Quick Inventory of Depressive Symptomatology; RSES, Rosenberg Self Esteem Scale; SCS, Self-Compassion Scale; WHO-HPQ, The World Health Organization Heath and Work Performance Questionnaire*.

## Discussion

### Overall Results

This is the first RCT of MBCT to feature a 2-month follow-up and to target the cognitive and affective aspects of SWB and eudaimonic well-being in community residents. Treatment adherence was high, and the absence of adverse events indicates the favorable feasibility of the program. In terms of the demographics of the participants, a higher proportion of participants were female, a general trend in other MBI studies (de Vibe et al., 2012).

Cognitive aspect of SWB score significantly improved by the end of the 8-week intervention. After 2 months' follow-up, the cognitive aspect of SWB improvement was further enhanced, and positive affect and eudaimonic well-being had also significantly improved.

For the secondary outcomes, most MAIA subscales showed significant improvements as early as 4 weeks. Previous research has shown that mind-body interventions increase awareness for interception (Bornemann et al., 2015) and awareness of interception is linked to mindfulness abilities (Hanley et al., 2017). Furthermore, it has been theoretically proposed that improved awareness of interception is the basis for metacognitive abilities fostered by mindfulness training (Garland et al., 2015). The present study's finding of significant improvements in awareness of interception followed by improvement of mindfulness skills accords with this suggestion. At 8 weeks, there was a significant improvement in mindfulness abilities and self-compassion, both of which are considered to be elements of the mindfulness mechanism (Kuyken et al., 2010; Gu et al., 2015). Further, resilience, self-esteem, and work productivity significantly improved at 16 weeks. This indicates that intensive mindfulness training can improve a variety of health outcomes, even with a reduced frequency of intervention after 8 weeks.

There was no significant improvement in some outcomes. In particular, anxiety and negative affect that were demonstrated to be improved by MBIs did not improve in the study (Schumer et al., 2018) (Ninomiya et al., 2019). In a study of Japanese undergraduate students, the mean score (SD) of the SPANE-N was 16.61 (4.87) (Sumi, 2014), which was similar to the results of the present study. Similarly, the mean score (SD) of the general population for GAD7 was 2.95 (3.41) (Löwe et al., 2008), which was similar to the score of the participants in the present study. Therefore, the participants in the study were mentally healthy individuals with low baseline anxiety and negative affect scores, which led to our inability to detect statistically significant improvement in the scales after the intervention.

### Relationship Between Mindfulness and Subjective Well-Being

Garland et al. (2015) proposed a mindfulness-to-meaning theory that explains the mechanism of the effect of mindfulness on well-being (Garland et al., 2015). This theory suggests that mindfulness practice increases metacognitive capacity for experience by improving mindfulness skills. This releases habitual perceptions of the experience, which leads to a broadening of recognition and a reconstruction of the experience. This results in a positive reappraisal of the experience and an increase in positive affect. Positive affect is linked to meaning in life through the recognition of experiences broadened by mindfulness training, which results in enhancing eudaimonic well-being. According to this theory, the order of improvement should be mindfulness skill, followed by the cognitive, affective, and eudaimonic well-being, respectively.

Interestingly, the temporal order in which mindfulness skill and each type of well-being improved in the study (i.e., the mindfulness skill and cognitive aspect of SWB after 8 weeks, followed by the affective aspects of SWB and eudaimonic well-being at 16 weeks) paralleled the order of improvement shown in the theory. Although more detailed research is needed, the framework of the theory could potentially explain the mechanisms by which each aspect of well-being improves.

### Clinical Implications

This study highlights a means of improving SWB and eudaimonic well-being among healthy individuals. Mindfulness is a “static” and “reflecting” intervention; therefore, it may represent a desirable option for people who do not prefer “activating” interventions (e.g., exercise, behavior activation).

### Limitations

First, the follow-up period, 2 months after the intervention, was relatively short. Second, although the participants were recruited from the community, because this was a single-center study with small samples, they didn't necessarily represent the characteristics of the whole population. Finally, the control group was a waiting group. Thus, we cannot exclude the influence of non-specific intervention effects. Future studies should include multi-center RCTs for more diverse community residents with active control groups and longer follow-up periods.

## Conclusions

In conclusion, we found that 8 weeks of MBCT with a 2-month follow-up period improves cognitive and affective aspects of subjective and eudaimonic well-being in healthy individuals. The order of improvement was cognitive, positive affective, and eudaimonic, respectively. Future studies should conduct multi-center RCTs with active control groups and longer follow-up periods.

## Data Availability Statement

The datasets presented in this article are not readily available because according to the ethics committee's rules, the Ethics Review Committee of the Keio University School of Medicine will review the request for release of the research data and, if permitted, we disclose it. Requests to access the datasets should be directed to Mitsuhiro Sado, mitsusado@keio.jp.

## Ethics Statement

The studies involving human participants were reviewed and approved by the Ethics Review Committee of the Keio University School of Medicine. The participants provided their written informed consent to participate in this study.

## Author Contributions

TK: Conceptualization, Data curation, Formal analysis, Investigation, Methodology, Project administration, Visualization, Writing—original draft, and Writing—review and editing. AN: Conceptualization, Data curation, Formal analysis, Investigation, Methodology, Project administration, Writing—original draft, and Writing—review and editing. MN: Investigation, Writing—review, and editing. ZH: Investigation and Writing—review and editing. KS: Investigation and Writing—review and editing. SP and DF: Conceptualization and Writing—review and editing. MM: Conceptualization, Supervision, and Writing—review and editing. MS: Conceptualization, Investigation, Methodology, Project administration, Supervision, Writing—original draft, Writing—review and editing, and Funding acquisition. All authors contributed to the article and approved the submitted version.

## Conflict of Interest

The authors declare that the research was conducted in the absence of any commercial or financial relationships that could be construed as a potential conflict of interest.

## Publisher's Note

All claims expressed in this article are solely those of the authors and do not necessarily represent those of their affiliated organizations, or those of the publisher, the editors and the reviewers. Any product that may be evaluated in this article, or claim that may be made by its manufacturer, is not guaranteed or endorsed by the publisher.
